# In Vitro Studies on a Microfluidic Sensor with Embedded Obstacles Using New Antibacterial Synthetic Compounds (1-TDPPO) Mixed Prop-2-en-1-one with Difluoro Phenyl

**DOI:** 10.3390/s17040803

**Published:** 2017-04-08

**Authors:** Changhyun Roh, Jaewoong Lee, Mayank Kinger, Chankyu Kang

**Affiliations:** 1 Biotechnology Research Division, Advanced Radiation Technology Institute (ARTI), Korea Atomic Energy Research Institute (KAERI), 1266, Sinjeong-Dong, Jeongeup, Jeonbuk 580-185, Korea; chroh@kaeri.re.kr; 2 Department of Textile Engineering and Technology, Yeungnam University, 280 Daehak-ro, Gyeongsan, Gyeongbuk 38541, Korea; jaewlee@yu.ac.kr; 3 Department of Chemistry, Maharishi Markandeshwar University, Mullana, (Ambala) Haryana 133207, India; mayank.kinger@yahoo.com; 4 Ministry of Employment and Labor, Major Industrial Accident Prevention Center, 34 Yeosusandallo, Yeosu-Si, Jeonnam 59631, Korea

**Keywords:** PDMS microfluidic sensor, confocal laser scanning microscope, 1-TDPPO, *Pseudomonas aeruginosa* PAO1, fluorescence intensity

## Abstract

This paper describes the use of an analytical microfluidic sensor for accelerating chemo-repellent response and strong anti-bacterial 1-(Thien-2-yl)-3-(2, 6-difluoro phenyl) prop-2-en-1-one (1-TDPPO). The chemically-synthesized antimicrobial agent, which included prop-2-en-1-one and difluoro phenyl groups, was moving through an optically transparent polydimethylsiloxane (PDMS) microfluidic sensor with circular obstacles arranged evenly. The response, growth and distribution of fluorescent labeling *Pseudomonas aeruginosa* PAO1 against the antimicrobial agent were monitored by confocal laser scanning microscope (CLSM). The microfluidic sensor along with 1-TDPPOin this study exhibits the following advantages: (i) Real-time chemo-repellent responses of cell dynamics; (ii) Rapid eradication of biofilm by embedded obstacles and powerful antibacterial agents, which significantly reduce the response time compared to classical methods; (iii) Minimal consumption of cells and antimicrobial agents; and (iv) Simplifying the process of the normalization of the fluorescence intensity and monitoring of biofilm by captured images and datasets.

## 1. Introduction

Microorganisms have the ability to detect or respond to specific chemical gradients [1]. Bacteria are influenced strongly by the types of chemicals used with respect to their colonization, infections of plants and animals, and signal recognition [2]. Bacteria that tend to agglomerate can resist small-molecule antibiotics and show 100 to 1000 times better survival than free-swimming cells [3,4]. Among the many types of bacteria, *Pseudomonas aeruginosa* PAO1 is a threat to human health because it tends to form agglomerates that encase themselves in the extracellular matrix of various polysaccharides and proteins [5]. As a result, the formed groups have strong adherence to and growth on surfaces, which leads to clinical infections. Furthermore, their strong resistance to antibiotic chemotherapy tends to make diseases more serious than in the past [6,7,8]. For example, bacteria can have a devastating effect on human life if they are not treated with strong antibiotics, because of their resistance to many antibiotics.

Several methods have been developed for the analysis of chemical interaction with bacteria, including agarose, collagen, stopped-flow diffusion chamber, and capillary assays [9,10,11,12]. These methods can enhance chemical delivery to microorganisms with the successful generation of chemo-effector gradients. These conventional methods, however, are limited due to (i) the difficulty in controlling the chemical gradient; (ii) fluctuations of the chemo-effector concentration; and (iii) the challenge of manipulating the chemical gradient [13]. To compensate for conventional methods, microfluidic devices have attracted considerable attention due to their actuated controls of micro-scale fluid flow with significant levels of automotive operation, enabling observations at high spatial and temporal resolutions in many microenvironments [14]. Thanks to the revolutionary advancement of micro-electronics fabrication and biotechnologies, microfluidic devices have become preferable choices in biological research [15,16]. Despite this, the diffusion of the chemo-effector with or without fluid flow in T-shaped micro-channels to achieve a linear chemo-effector gradient has similar limitations of the temporal stability of the chemical gradient to conventional methods [17,18]. In order to improve the mixing performance, a simply narrower or long and arbitrarily-shaped microchannel is used to induce high shear stress and diffusion, but the performance of these is not outstanding [19,20,21]. Recently, circular-shaped micro-posts have been widely used in electronic devices for rapid cooling with liquid solutions by increasing vortex-induced fluctuation, and these show outstanding mixing performance [22,23]. An appropriate array of micro-posts can lead not only to improved contact with the medium, but also to a flow-induced chaotic flow for enhanced mixing performance compared to a simple microfluidic channel with higher shear stress [24]. Microfluidic devices integrated with embedded obstacles, which have been used as passive micro-mixers, offer the following advantages over plain microfluidic devices: (i) enhanced heat and mass transfer coefficients [25,26]; (ii) increased mixing efficiency by reducing diffusion path [27,28]; and (iii) allowance of chemical gradient [29,30]. Therefore, the optimal design for a microstructure-integrated microchannel assembly will increase its efficiency in microfluidic systems. In recent years, the combination of a microfluidic system and confocal laser scanning microscope (CLSM) has also been used widely as a supplementary method for evaluating chemical-to-biofilm formation and eradication [31,32,33,34]. It directly allows for the visualization of cell distribution with fluorescent labeling, and the analysis of quantifying biofilm was proportional to fluorescence intensity, which is considered as an effective means [35,36].

The purpose of this study is to focus on the fact that the newly invented 1-TDPPO is superior to the existing chemical agent (i.e., ethanol), and this effect is in turn enhanced by the microstructures embedded in the microfluidic system. 1-TDPPO, a newly synthesized antimicrobial agent, was used and based on the fact that prop-2-en-1-one or difluoro phenyl exhibited a very strong antimicrobial efficacy [37,38,39]. A microfluidic sensor, which offers spatial and temporal profiles, observes the growth and eradication with a controllable manner. To understand the importance of the design of the microfluidic sensor, we conducted a study on the antibacterial properties of 1-TDPPO with or without circular obstacles embedded on the microfluidic sensor. Throughout this study, CLSM was utilized as an accurate analysis technique by achieving 3-dimensional images. A real-time monitoring between fluorescent labeling *Pseudomonas aeruginosa* PAO1 and an antimicrobial agent was observed directly by a fluorescence intensity surface plot, providing information on the bacterial distribution from achieved images inside the microfluidic reactor. A stable biofilm growth and a quick response of the antimicrobial agent were induced by artificially-embedded obstacles using the volume analysis method. The growth and eradication of biofilm was a major determinant for the antimicrobial efficacy, whereas the efficacy of microfluidic sensor was determined by the response time.

## 2. Results and Discussion

### 2.1. Selection of Antimicrobial Concentration

**Figure 1:**
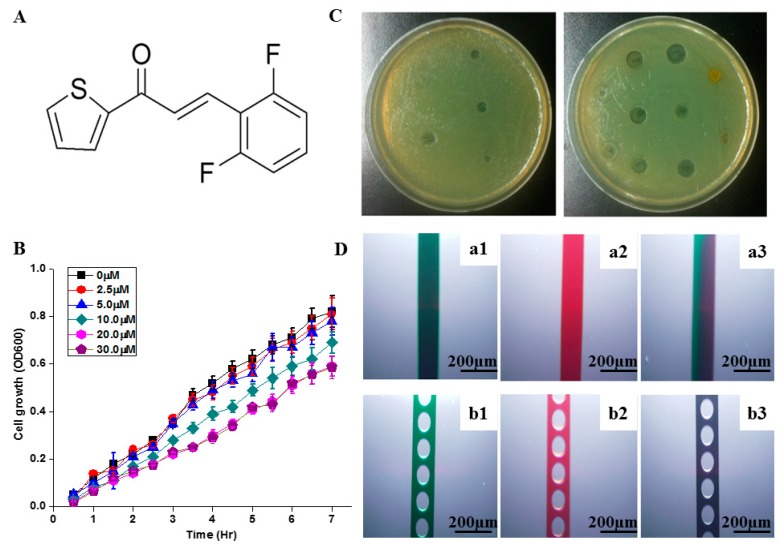
(**A**) Chemical structure of 1-(Thien-2-yl)-3-(2,6-difluoro phenyl) prop-2-en-1-one (1-TDPPO); (**B**) chemical response of the antimicrobial agents (20 µM of 1-TDPPO) at day 1 (left) and day 3 (right) in a Petri dish; (**C**) selection of the optimal concentrations for the antimicrobial effect with 1-TDPPO; and (**D**) comparison of mixing efficacy between without (**a1**–**a3**)/with (**b1**–**b3**) circular obstacles using green and red dyes. Here a3 and b3 are images after mixing.

1-TDPPO was examined by adding the synthesized compound by inoculation to reach final concentrations of 0, 2.5, 5, 10, 20, and 30 µM to determine the ranges of concentrations. Figure 1A shows the chemical structure of synthetic 1-TDPPO. The nuclear magnetic resonance (NMR) data of 1-TDPPOwas: ^1^H NMR (500 MHz, CDCl_3_, δ, ppm): 6.96 (2H, m), 7.18 (1H, dd, *J* = 4.8, 3.7 Hz), 7.32 (1H, m), 7.71 (2H, m), 7.85 (1H, dd, *J* = 3.7, 1.1 Hz), 7.91 (1H, d, *J* = 16.0 Hz); ^13^C NMR (125 MHz, CDCl_3_, δ, ppm): 111.9, 127.2, 128.3, 129.9, 131.2, 132.2, 134.3, 145.4, 161.0, 163.0, 182.2. All of the compounds were added directly to the growth medium at the time of inoculation. As shown in Figure 1B, representative growth curves showed that the cell growth of PAO1-GFP was inhibited by 1-TDPPO in a dose-dependent manner. Compared to the control sample, the specific growth rates of PAO1-GFP in the presence of 1-TDPPOat 2.5, 5, 10, 20, and 30 μM were reduced by 1.2%, 4.9%, 15.9%, 28.0%, and 27.9%, respectively. In the case of 30 μM, the antimicrobial efficacy did not increase significantly compared to 20 μM and there was a problem in the synthesis process with more than 30 μM. Therefore, a concentration of 1-TDPPO at 20 μM was chosen as an optimal inhibitory concentration for biofilm growth and exhibited strong cell response and biofilm eradication using the microfluidic system. Killing or leveling-off of bacterial growth was demonstrated in Figure 1C. The experiment, performed in a Petri dish-based system, indicated that the antimicrobial effect with 1-TDPPO with an aryl moiety had a strong influence on *Pseudomonas aeruginosa* PAO1, which encased themselves in the extracellular matrix of various polysaccharides and proteins. This result indicated that it took three days to confirm its strong antimicrobial property, despite its outstanding antimicrobial properties. In the case of the Petri dish system, molecular diffusion, the dominant mixing process, proceeds relatively slow and inhibits efficient mixing. This simply demonstrates the superiority of our microfluidic system, which induced a fast response compared to a three-day reaction time. The mixing performance by embedded circular microstructures was compared by microchannel without microstructures in Figure 1D. These mixing tests with dyes were shown that embedded microstructures induced complete mixing. The microfluidic system reduced the mixing time by decreasing the length scale and embedding microstructures. 

### 2.2. Real-Time Monitoring of Cell Dynamics

**Figure 2:**
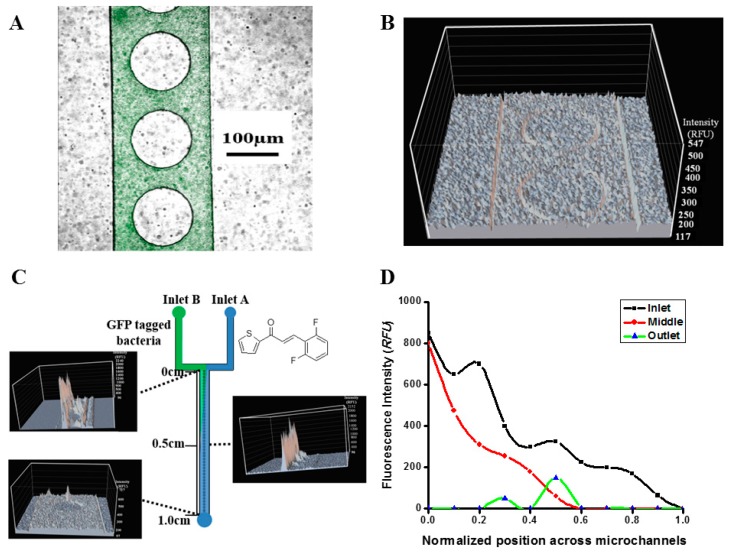
(**A**) Actual image of *P. aeruginosa* PAO1 inside microfluidic sensor without 1-TDPPO; (**B**) fluorescence intensity profile after *P. aeruginosa* PAO1 reacted with 1-TDPPO; (**C**) antimicrobial activity based on GFP-tagged fluorescence intensity (inlet A: 1-TDPPO; inlet B: *P. aeruginosa* PAO1); and (**D**) the fluorescent intensity (RFU) along with the normalized microfluidic channel position.

After determining the concentration of and injecting the 1-TDPPO, the real-time cell dynamics were observed. Figure 2A presents the image of the *P. aeruginosa* PAO1 passing through a microfluidic chip, and the actual observed the pixel intensity (R.F.U.) was 233 without 1-TDPPO. Strong fluorescence intensity was observed when *P. aeruginosa* PAO1 moved throughout the microfluidic sensor. This means that the microfluidic sensor can be used to observe the real-time response of bacteria against the antimicrobial agent. In contrast to Figure 2A, no fluorescence was observed when 1-TDPPO had passed completely through the microfluidic sensor, as shown in Figure 2B. The real-time monitoring of the *P. aeruginosa* PAO1 activity performed at three different positions (i.e., inlet, middle and outlet), as shown in Figure 2C, revealed different characteristics. The strong chemo-repellent behaviors away from 1-TDPPO were observed in the channel outlet. Therefore, high fluorescence intensity was initially observed in the channel inlet, whereas this phenomenon became weaker as the fluid-flow approached the channel outlet, indicating that this system can be produced with user-defined gradients of spatial and temporal profiles in a predictable and controllable manner. This meant that the mixing did not occur well at the inlet, but the mixing was smooth as it approached the outlet. As a result, the fluorescence intensity decreased as the cell density decreased. The analysis of fluorescence intensity was normalized as a function of the position, as shown in Figure 2D. This means that a large number of GFP-tagged *P. aeruginosa* PAO1 bacteria were located in the inlet, whereas there was a small number of bacteria in the outlet due to the antimicrobial agent. Throughout this process, the passive chemo-repellent gradient could be achieved within a very short period of time using a simple method because the embedded circular obstacles in the microfluidic reactor led to enhanced mixing efficiency without requiring a gradient generator. This device for real-time monitoring of cell dynamics can also be reused after washing.

### 2.3. Biofilm Formation in Microfluidic Sensor

**Figure 3:**
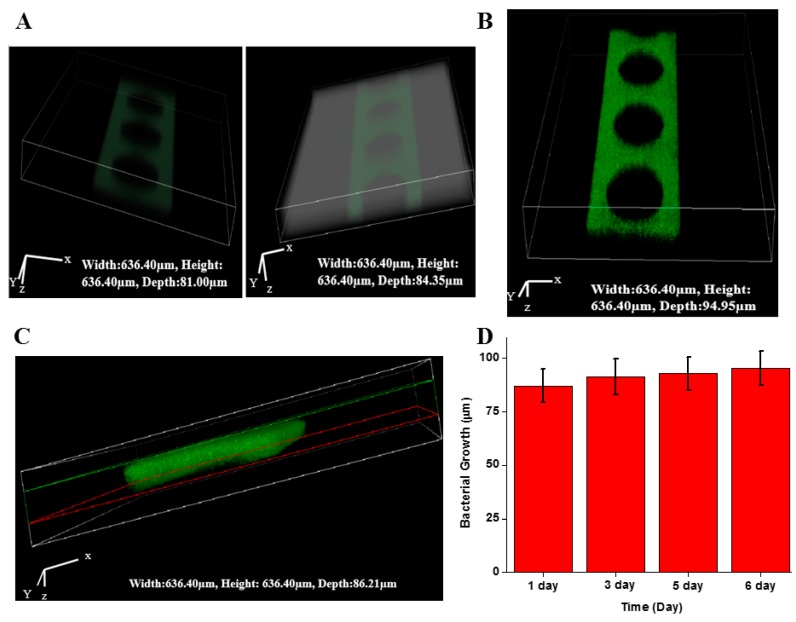
Analysis of biofilm formation in the microfluidic sensor. (**A**) 3D images after 12 h (left) and 24 h (right); (**B**) 3D image after 6 days; (**C**) analysis of *P. aeruginosa* biofilm thickness using confocal microscopy (red line indicates the actual biofilm thickness); and (**D**) time-dependent analysis of biofilm thickness.

Bacteria aggregates began to be detected at 12 h and biofilm growth became significant within 24 h, as shown in Figure 3A. This proved that biofilm was successfully observed and grew at certain periods. However, the growth of the *P. aeruginosa* PAO1 biofilm appeared to be relatively constant from 24 h until day 5, as the three-dimensional profiles of the fluorescence intensity did not provide a characteristic difference. The images shown in Figure 3B were observed as a three-dimensional CLSM image which clearly showed that there were a large number of cell clusters on day 6. A strong fluorescence intensity compared to the images in Figure 3A was detected. To analyze the direct measurements of the biofilm formed, three-dimensional volume analysis was performed on CLSM with the selection of the top and bottom of the polydimethylsiloxane (PDMS) structure, as shown in Figure 3C. Such a direct measurement suggests a convenient and economic means, as many studies have used numerical models and extra software programs to predict biofilm thickness. The growth of the biofilm with a signal of increasing fluorescence intensity, as shown in Figure 3B, was observed on day 6, after several days of stagnation. Figure 3D presents the results of a statistical analysis of the biofilm thickness with error bars that were generated by the heterogeneity of *P. aeruginosa* PAO1 biofilm existing on the microfluidic chip based upon position. The difference in the biofilm thickness was less than 5% in all cases. From the given data, an analysis of the biofilm in microfluidic sensors along with CLSM can be considered a reliable method and is expected to increase the use of this system.

### 2.4. Biofilm Eradication in Microfluidic Sensor

**Figure 4:**
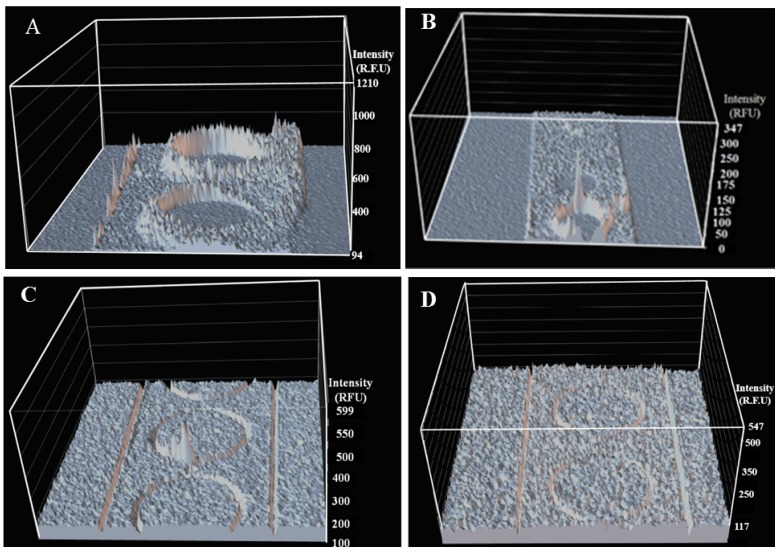
An analysis of *P. aeruginosa* PAO1 biofilm with two different antimicrobial agents. (**A**) 20% ethanol solution with a 30-min mixing time; (**B**) 90% ethanol solution with a 7-min mixing time; (**C**) 1-TDPPO with a 5-min mixing time; and (**D**) 1-TDPPO with a 7-min mixing time.

The real-time cell monitoring of 1-TDPPO provided a clue that it can be useful in eradicating the biofilm. The efficacy of 1-TDPPO for the eradication of *Pseudomonas aeruginosa* PAO1 was investigated and compared with those of 20% and 90% ethanol solutions, as shown in Figure 4. Ethanol is a typical chemical used as an antimicrobial agent for bacteria, and several studies have been published to suggest its efficacy [40]. Therefore, a comparative study with ethanol was conducted to demonstrate the superiority of 1-TDPPO. The reason for selecting the aforementioned range was that a low concentration of 20% ethanol difluoro has been known to result in all cells embedded in the biofilm being killed. However, the concentration of 60 to 90% ethanol was also considered as an optimal antimicrobial concentration [40,41,42]. The analysis result shown in Figure 4A indicates that the formed biofilms were not eradicated completely by exposure to a 20% ethanol solution after a 30-min mixing time. It took 45 min to completely eradicate the biofilm, which exhibited outstanding performance in comparison to the microplate system [40]. Increasing ethanol concentration up to 90%, as shown in Figure 4B, significantly reduced the eradication time for the biofilm due to the high concentration of the classical antimicrobial agent. However, it required about 10 min due to their strong adhesions to the microchannel surfaces and microstructure walls. The existence of several bacterial residuals in the form of fluorescent intensity comes from a few plausible reasons. One is that the relatively rough surface caused by the fabrication of the microfluidic sensor could interrupt the bacteria eradication due to the strong cell attachment [43]. However, the surface roughness measured in this study was less than 20 nm, which significantly reduced cell attachment on the surface, but residual bacteria still provided a critical negative effect on the outcome of sensor performance. Another reason, based on a recent study, could be the insufficient mixing time [44,45]. Even though a microfluidic sensor with regularly embedded obstacles showed an increase in mixing efficacy, it did not perform well under the present conditions. Eventually, increasing the mixing time to 30 min showed a significant improvement in the eradication of *Pseudomonas aeruginosa* PAO1 and totally removed them. In addition, the increasing concentration of antimicrobial agent (i.e., 90% ethanol) resulted in a better performance. Tiny residuals of *Pseudomonas aeruginosa* PAO1 were present in the microfluidic sensor when it was operated for 5 min with 1-TDPPO, as shown in Figure 4C. Finally, the remaining *Pseudomonas aeruginosa* PAO1 finally disappeared when the mixing time was increased to 7 min, as shown in Figure 4D. The mean cell density obtained by confocal microscopy, as shown in Figure 4B,C, was 411 ± 33 and 282 ± 21, respectively. The intensity surface plot expressed as three-dimensional images showed spiked peaks, which represented the remaining groups of bacteria. Because very small residuals were not observed by the naked eye and volume analysis in some cases, the use of an intensity surface plot in CLSM was required. As a result, it can safely be concluded that the combination of the current microfluidic sensor and 1-TDPPO may be effective in rapidly eradicating biofilms compared to a classical solution. The embedded circular obstacles exhibited a better mixing performance compared to the absence of microstructures under the same experimental condition. Table 1 shows the change of fluorescence intensity according to various factors. The images observed during the mixing time of 7 min indicated, as shown in supplemental data, that the selection of antimicrobial agents and microfluidic designs are important factors in biofilm eradication. On the other hand, the absence of microstructures lead to relatively high fluorescence intensity. When mixing time was applied for 7 min, the comparison of fluorescence intensity with other chemical agents at the microchannel outlet and the presence or absence of microstructures is shown in supplemental data. 

## 3. Materials and Methods

The bacterial strain used in this study was the *P. aeruginosa* PAO1 wild-type strain. The bacteria were transformed by electroporation with a plasmid, which stably expressed a green fluorescent protein (GFP). *P. aeruginosa* PAO1 was grown in a Luria–Bertani (LB) medium supplemented with carbenicillin (50 μg/mL) for recombinant selection and retention. The stationary phases used for column chromatography (Silica gel 60, 70–230 mesh) and thin-layer chromatography (TLC) plates (Silica-gel 60 F254) were purchased from Merck KGaA. All other chemicals were obtained from Sigma-Aldrich (St. Louis, MO, USA). The final concentration of bacteria in this study was 1 × 10^9^ cells/mL.

### 3.1. Synthesis of Antimicrobial Agent (1-TDPPO)

Aqueous sodium hydroxide (0.011 Mol) at 0 °C was added to a mixture of 2-acetylthiophene (1 mL, 0.0092 mol) and 2,6-difluorobenzaldehyde (1.72 g, 0.0092 mol) in ethanol. The solution was stirred for 6–8 h at room temperature. Upon completion of the reaction according to TLC, the reaction mixture was poured into ice-cold water, and the pH of the mixture was then adjusted to 6 by adding 0.01 N HCl solutions. The obtained precipitates were filtered, dried and re-crystallized from the mixture to obtain the compound. The melting points were determined in open capillary tubes and were uncorrected. The ^1^H nuclear magnetic resonance (NMR) and ^13^C NMR spectra were recorded on a JNM-ECA 500 spectrophotometer (Jeol) at 500 and 125 MHz, respectively. The internal standard used for these spectra was tetramethylsilane (TMS). The IR spectra (KBr) were recorded on a Bruker-Vector 22 instrument. The molecular mass of the compounds was derived from the high-resolution mass spectral (HRMS) technique using a JEOL JMS600 instrument.

### 3.2. Antimicrobial Activity Using Petri Dish System

The anti-bacterial activity was pre-examined using the halo method on a solid agar plate. *P. aeruginosa* PAO1 was seeded onto a solid agar plate using the spread plate method. 10 μL (10^6^ cell/mL) of *P. aeruginosa* PAO1 was cultured at 37 °C for 24 h to induce cell growth in the solid medium. The compounds were placed on a tested bacteria-seed plate. The anti-bacterial assay plate was again incubated at 37 °C for 24 h. The solvent without the compounds was used as a control sample to compare the antimicrobial efficacy.

### 3.3. Fabrication of Microfluidic Reactor

A microfluidic chip with/without circular obstacles in the micro-channels was fabricated using standard photoresist-based soft lithography [46]. A positive photoresist (AZ P4620) was applied to a 4-inch silicon wafer (Silicon Quest International, Santa Clara, CA, USA). A mold, 5 mm in height, was placed on the wafer surrounding the photoresist layer and PDMS (GE RTV 615 elastomer: cross-linker = 10:1) was then poured onto the wafer inside the mold structure to produce a 5 mm-thick chip, which was then cured at 80 °C for 1 h. The microfluidic reactor was peeled off the wafer/photoresist and the holes for inlet and outlet ports were fabricated using a 19 gauge punch (Technical Innovations, Inc., Brazoria, TX, USA). A plasma cleaner was used to remove the impurities from the surface of the PDMS structure and glass slide, and a final thermal bonding was treated at 80 °C for 18 h. The fabricated microfluidic reactor had a measured width equal to 243 ± 1 μM and a measured height of 100 ± 5 μM. The diameter of the microstructures used was 152 μM. An aligned row of periodic obstacles was arranged along the centerline of each channel. The micro-fabricated obstacles in this study had diameters of 132 μM. The mean surface roughness of the microfluidic sensor, measured using a surface profilometer (Veeco instruments, Inc., Plainview, NY, USA), was less than 20 nm.

### 3.4. Fluorescence Bio-Imaging

The analyses of both the antimicrobial activity and population of bacteria were measured by Nikon Eclipse Ti-E spinning disk confocal microscopy (Nikon, Japan). Fluorescence was stimulated at a 488 nm wavelength, and a filter isolated the emitted wavelength between 505 and 539 nm, which is the green portion of the visible spectrum [47]. A series of images with a Z-scan were compiled using objective lenses of 10x and 40x to determine the thickness of the PAO1-GFP biofilm by volume measurements. The three-dimensional image files generated by the Z-scan were available to determine the top and bottom of the biofilm. The level of the biofilm was observed in the bottom of the images, which included detailed information on the actual width, length, and depth without complicated works. In addition, the real-time fluorescence intensity, which indicates bacterial distributions, was monitored using an intensity surface plot in CLSM (X axis: position, Y axis: pixel). This reduces the complicated process for the normalization of fluorescence intensity. During image processes, the focal-plane depth, laser power, and signal gain were maintained at constant values.

### 3.5. Experimental Procedures

**Figure 5:**
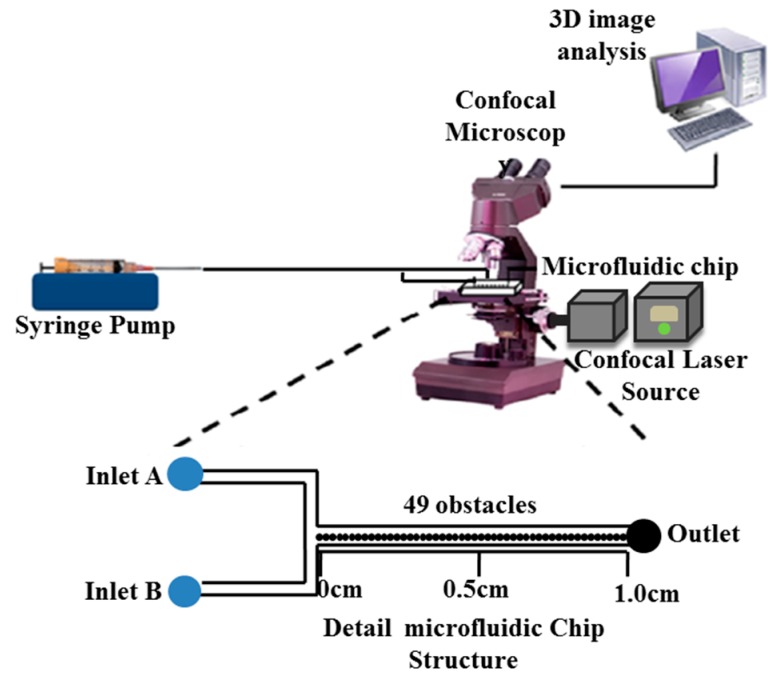
Experimental setup for bacterial activity, biofilm growth and eradication with a microfluidic sensor with microstructures.

Two Tygon tubings (0.06”OD × 0.0200”ID, Saint-Gobain Corp., Akron, OH, USA) were pre-cleaned with ethanol and distilled water before being connected by a syringe pump (Harvard Apparatus, Holliston, MA, USA) to the microfluidic chip at the two microfluidic chip inlets, as shown in Figure 5. 

The prepared *P. aeruginosa* was introduced into inlet A and the synthetic antimicrobial agent was injected into inlet B. All experiments were conducted at room temperature. To observe a real-time dynamic response of cells against the antimicrobial agent, the employed flow rate was 0.5 μL/min using a syringe pump (±0.5% accuracy) to observe a chemo-repellent gradient within the microfluidic sensor. The optimal selection of the flow rate is the limiting point of this technique. We performed the chemical gradient on injecting 1-TDPPO at flow rates of 0.05 µL/min to 5.0 µL/min and selected the optimal flow rate of 0.5 µL/min. The criteria used were a compromise between high flow rates that occasionally interrupt the bacterial motility, and low flow rates that do not obtain a linear chemical gradient. As a result, preliminary tests were performed on the microfluidic channels, and the optimal flow rate, 0.5 μL/min, was selected. The bacterial responses and their distribution profiles through the microfluidic sensor were observed directly by CLSM. A three-dimensional image of the fluorescence intensity profile was proportional to the metabolically-active population of bacteria which applied the principle as two-dimensional bacterial responses [48]. Throughout this process, the bacterial distribution and chemo-repellent gradient can be observed in the microfluidic reactor as a function of time and position. *Pseudomonas aeruginosa* PAO1-GFP in the microfluidic system was incubated at 37 °C for 7 days before the determination of colony-forming unit (CFU) per mL. Luria-Bertani (LB) media and *Pseudomonas aeruginosa* PAO1 were initially introduced to the two inlets until the microfluidic reactor was filled. The formation of the *Pseudomonas aeruginosa* PAO1 biofilm was monitored and analyzed continuously by CLSM using the volume analysis method. After biofilm formation, deionized water was injected into the microfluidic channels to remove the dead bacteria. 1-TDPPO was then introduced into inlets at 0.5 μL/min. The change of biofilm was also measured by CLSM. To compare the efficacy of 1-TDPPO, popular antimicrobial agents, 20% and 90% ethanol, were used in this study. It was known that a low concentration of 20% ethanol demonstrated that all cells embedded in the biofilm were killed [40]. According to the CDC (Centers for Disease Control and Prevention), ethanol at concentrations of 60%–80% is considered as a potent virucidal agent, inactivating all of a virus [49,50]. Another study claimed that the range of 60% to 90% ethanol was an optimal concentration for killing the biofilm [42,51]. Under these experimental conditions, the microfluidic sensor was operated at very low Reynolds numbers (10^−4^~10^−3^), in which the antimicrobial agent was transported to the surface by diffusion. 

## 4. Conclusions

This study proposed and examined the potential use and merits of microfluidic sensors for the direct analysis of cell response, biofilm formation and eradication of *P. aeruginosa* PAO1 using a new synthetic chemical, 1-TDPPO, which has organic compounds of prop-2-en-1-one and difluoro phenyl. The combination of two organic compounds exhibits a stronger antimicrobial efficacy than the more commonly used antibacterial agents. The bacterial distribution in response to antimicrobial agents was observed directly by CLSM using the intensity surface plot, which provided a high-throughput chemo-repellent gradient. Microfluidic sensors for generating gradients offer spatial and temporal profiles in a predictable and controllable manner and can be compensated using conventional methods. A rapid response of cell dynamics in microfluidic sensors, which provides information about cell response, was directly observed. It eventually saved a lot of processing time for the normalizing of fluorescence intensity, which was used by the software program. It was clear that *P. aeruginosa* PAO1 exhibited a strong tendency to repel 1-TDPPO in the channel inlet and was depleted completely in the channel outlet. *P. aeruginosa* PAO1 was formed in clusters within 24 h and exhibited a significant change in fluorescence intensity in 6 days, as observed using microfluidic sensors. The growth of the biofilm was observed by CLSM using a three-dimensional volume analysis method, which replaced the numerical models for measuring the depth of the biofilm. The microstructures embedded in the flow-based microfluidic sensor showed superior antibacterial activity compared to no microstructures. Using a microfluidic sensor embedded in microstructures and synthetic 1-TDPPO, the biofilm was eradicated in a short time and exhibited outstanding antimicrobial efficacy. 20% and 90% ethanol solutions led to successful performances of eradicating biofilm formation, but sufficient mixing time should be considered in advance.

## Figures and Tables

**Table 1:** Summary of fluorescence intensity (RFU) with antimicrobial agents.

	0 min	5 min	7 min	10 min	30 min	40 min
1-TDPPO (20 µM) with microstrctures	771 ± 44	196 ± 16	0	0	0	0
1-TDPPO (20 µM) without microstrctures	731 ± 39	512 ± 33	357 ± 28	258 ± 20	0	0
Ethanol (20%) with microstrctures	753 ± 48	531 ± 26	433 ± 25	366 ± 23	211 ± 12	0
Ethanol (20%) without microstrctures	767 ± 57	681 ± 48	598 ± 34	511 ± 43	458 ± 37	0
Ethanol (90%) with microstrctures	758 ± 36	302 ± 17	178 ± 13	0	0	0

## Supplementary Materials

The Supplementary Materials are available online at www.mdpi.com/, Figure S1: Analysis of fluorescence intensity with various cases. Here, mixing time was 7 minutes. (**A**) Ethanol (20%) without microstructures, (**B**) 1-TDPPO without microstructures, (**C**) ethanol (20%) with microstructures, (**D**) ethanol (90%) with microstructures.
