# Supplementary material for: In Vitro Studies on a Microfluidic Sensor with Embedded Obstacles Using New Antibacterial Synthetic Compounds (1-TDPPO) Mixed Prop-2-en-1-one with Difluoro Phenyl
Source: Sensors (Basel). 2017 Apr 8;17(4):803. doi: 10.3390/s17040803 (PMC5422164; doi:10.3390/s17040803)
Supplement: Supplementary file 1 [file sensors-17-00803-s001.pdf]

Supplementary Materials: In Vitro Studies on a Microfluidic Sensor with Embedded Obstacles Using New Antibacterial Synthetic Compounds (1-TDPPO) Mixed prop-2-en-1-one with Difluoro Phenyl

Changhyun Roh, Jaewoong Lee, Mayank Kinger and Chankyu Kang

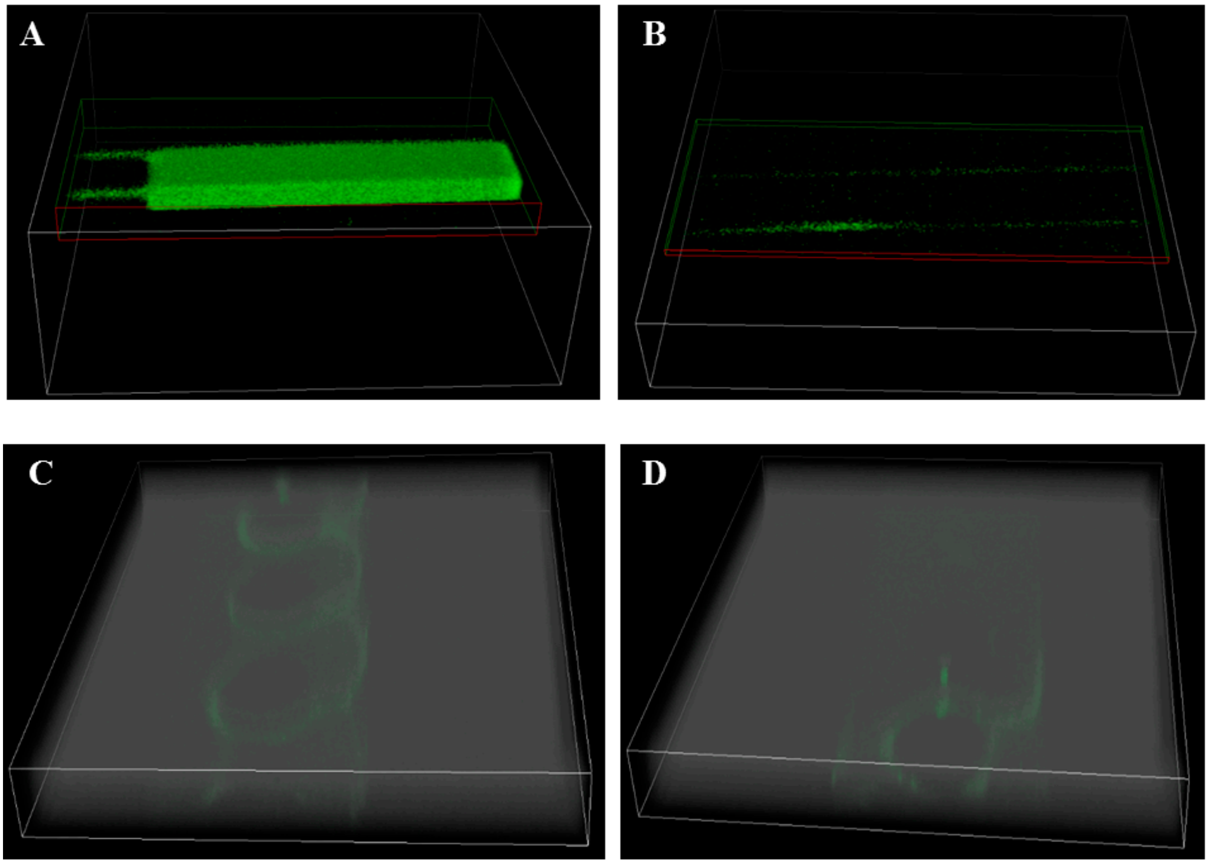

Supplemental Data 1. Analysis of fluorescence intensity with various cases. Here, mixing time was 7 minutes. (a) Ethanol (20%) without microstructures, (b) 1-TDPPO without microstructures, (c) ethanol (20%) with microstructures, (d) ethanol (90%) with microstructures.
